# Supplementary figures and images for: Aggregatibacter actinomycetemcomitans Induces Autophagy in Human Junctional Epithelium Keratinocytes
Source: Cells. 2020 May 14;9(5):1221. doi: 10.3390/cells9051221 (PMC7290389; doi:10.3390/cells9051221)

Figure S1

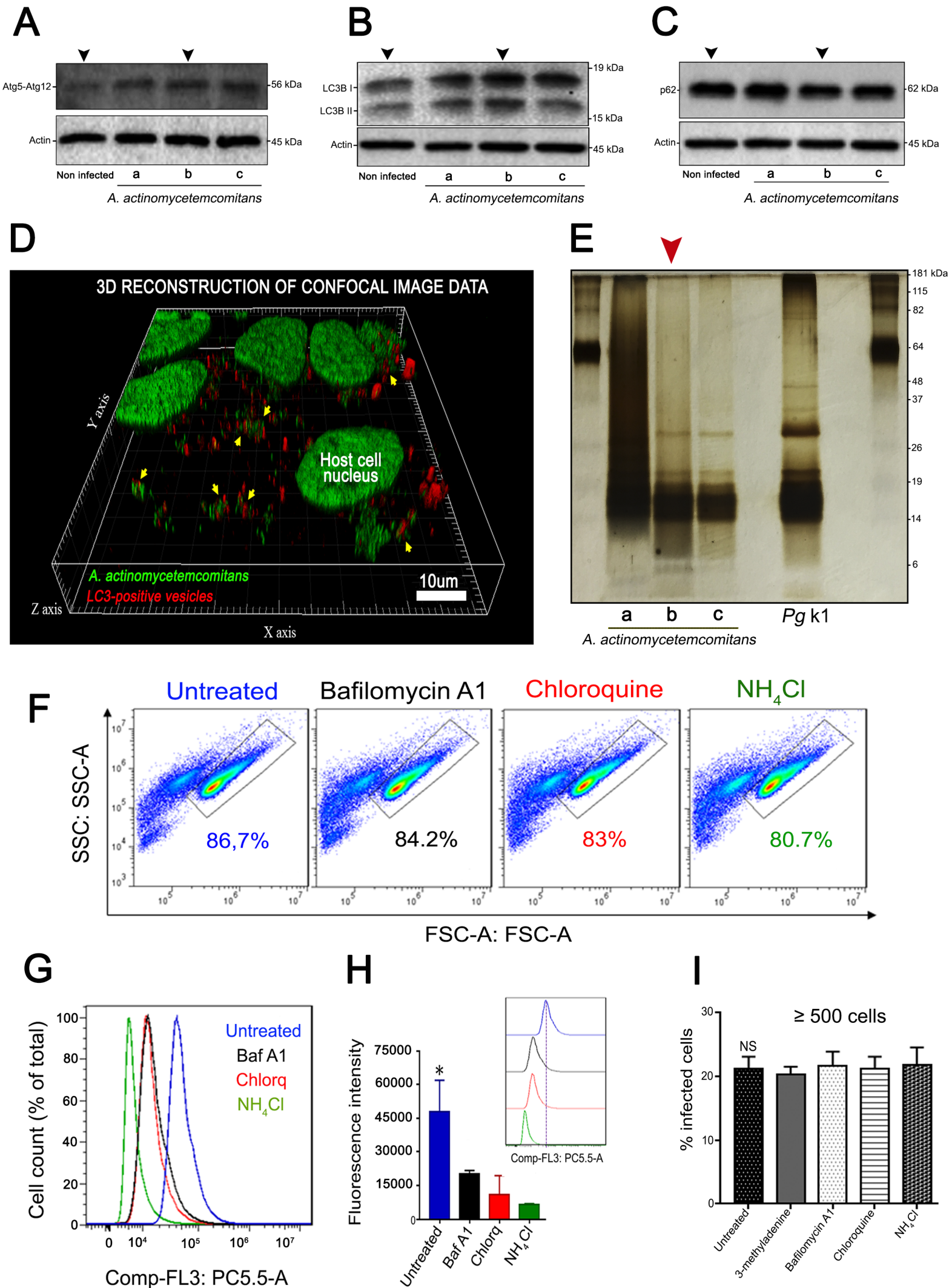

Supplement: Supplementary file 1 [file cells-09-01221-s001.pdf]
